# Limits of data anonymity: lack of public awareness risks trust in health system activities

**DOI:** 10.1186/s40504-021-00115-9

**Published:** 2021-07-26

**Authors:** Felix Gille, Caroline Brall

**Affiliations:** Department of Health Sciences and Technology, Ethics and Policy Lab, ETH Zürich, Zürich, Switzerland

**Keywords:** Anonymity, Identifiable data, Trust, Privacy protection, Data literacy

## Abstract

Public trust is paramount for the well functioning of data driven healthcare activities such as digital health interventions, contact tracing or the build-up of electronic health records. As the use of personal data is the common denominator for these healthcare activities, healthcare actors have an interest to ensure privacy and anonymity of the personal data they depend on. Maintaining privacy and anonymity of personal data contribute to the trustworthiness of these healthcare activities and are associated with the public willingness to trust these activities with their personal data. An analysis of online news readership comments about the failed care.data programme in England revealed that parts of the public have a false understanding of anonymity in the context of privacy protection of personal data as used for healthcare management and medical research. Some of those commenting demanded complete anonymity of their data to be willing to trust the process of data collection and analysis. As this demand is impossible to fulfil and trust is built on a false understanding of anonymity, the inability to meet this demand risks undermining public trust. Since public concerns about anonymity and privacy of personal data appear to be increasing, a large-scale information campaign about the limits and possibilities of anonymity with respect to the various uses of personal health data is urgently needed to help the public to make better informed choices about providing personal data.

## Introduction

Common public and professional agreement exists, that high levels of trust are critical for the well-function of data driven activities within health systems (Lawler et al. 2018). Sztompka defines trust as “a bet about the future contingent actions of others” (Sztompka 1999, 25). In data driven activities, trust is a relational construct where data donors trust the health system with their data in anticipation of a net-benefit for the health system, the donor and society (Gille et al. 2020). Examples of such trust relationships are the build-up of electronic health records to be able to improve health management and quality of care (Hays and Daker-White 2015), the collection of data to curb the coronavirus 2019 pandemic (Ienca and Vayena 2020), donation of personal data and biospecimens to biobank facilities for personalized health research (Brall et al. 2021), or acceptance of digital health interventions in general (Brall et al. 2019). As the use of personal data is the common denominator for these health system activities, all of them have an interest to ensure privacy and anonymity of the personal data they depend on. Recognising that both are contested concepts, we understand anonymity as “… one has anonymity or is anonymous when others are unable to relate a given feature of the person to other characteristics” (Wallace 1999, 24). Wallace’s definition of anonymity fits well to the issue discussed in the article as Wallace’s work on anonymity centers around public perceptions, social interactions and information systems. Ostherr and colleagues describe privacy as “health data privacy is not a stable natural object that has value regardless of the subjects who enact it; rather, health data privacy is a multifaceted cultural artifact that becomes assembled and maintained within a complex ecology of alliances and disconnections.” (Ostherr et al. 2017, 6). In contrast to set in stone definitions of privacy, one could follow Nissenabum and understand privacy as contextual integrity where privacy is defined by appropriate information flows in a given context. Appropriateness is shaped by the norms and values of the context. The context is described by a set of parameters: data subject; data sender; data recipient; information type; and transmission principle (Nissenbaum 2010). Further, privacy is controlled by existing oversight systems, laws and regulation and not something much in control of individuals (Ballantyne 2020). Hence, many individuals consider trust in data users and institutions to maintain anonymity and privacy as a critical issue.

Research shows that concerns about privacy are affecting patients willingness to share their medical history (Walker et al. 2017). For donors to be able to make an accurate decision about their willingness to share medical data and to build trust in health actors using the data, a precise understanding of what data anonymity means is essential. Otherwise, a decision to share data and subsequently donor’s trust is based on false assumptions.

Unfortunately, we observed in previous research on public trust in the English health system a tension between the limits of data anonymity and the rigorous expectations of parts of the public that their data have to be completely anonymised throughout its use both for the analysis of health service performance and medical research (Gille et al. 2020). While a popular understanding is that full anonymity should be guaranteed in all circumstances, the understanding in the research arena is that full anonymity is not feasible. From a research perspective there is the need to be able to link different data sets as the most value is to be made of linked personal health data in the public interest, namely for scientific discoveries. The values associated with data linkage are many, as for example, increased accuracy in long-term studies; the possibility to have a treatment and control group in one data set; being able to research the impact of the environment on health where environmental data is linked with health data; or researching rare diseases in large data sets (Wellcome Trust 2015). The observed tension threatens public trust in the health system, as the public understands the preservation of data anonymity as an important element for public trust in the health system. In this article we aim to highlight our observation and to discuss possible remedies.

## Main text

### Members of the public demand complete anonymity, but science recognizes the limits of anonymity in times of linking health data

As part of a research project on public trust in the health system, a secondary analysis of British national online news articles (published between 2013 and 2015) with their readership comments about the NHS’ care.data programme (*Articles*: British Broadcasting Corporation *n = 2;* Daily Mail *n = 16;* Guardian *n = 14;* Independent *n = 15;* Telegraph *n = 11. Readership comments total*: *n = 1625*) identified the importance of data anonymity for public trust in the health system (Gille et al. 2020). Care.data aimed to link and share general practitioner and hospital data about individuals’ care to improve the quality of care for all. Yet the programme failed as a result of public concerns about the ability to keep sensitive information secure and the potential for commercial gain to be made from patients’ personal data (Carter et al. 2015; NHS 2014). In a nutshell “The programme aimed at securing the bare minimum of trust while maximising potential returns on investment. It thus quickly dismissed privacy and respect for individual autonomy as individualistic rights opposing wider prosperity, rather than seeing them as principles of social trust and public engagement“ (Vezyridis and Timmons 2019) The rationale for the choice of news outlets was to achieve national coverage. News articles covering care.data were searched via Google.com or search engines on the news webpages in 2015. Readership comments were downloaded and analysed inductively according to a thematic analysis approach with NVIVO 9.

From a wide range of readership comments the following direct quotes from the online news readership comments sections show how parts of the public understand the role of anonymity for trust in the care.data programme and wider health system. Without doubt, other readership comments suggest that other parts of the public understand the limits of anonymity. At the time, news articles in England discussed the possible introduction of the care.data programme and what its implications are for the NHS England. Mirroring the critical voice of many professional associations and public voice the majority of articles was written with a critical if not negative tone towards the care.data programme.

*… Destroy our faith and trust … and there will be no return. The only way to do this is either absolute and unbreakable anonymity or not at all …*

Comment on: (Chapman and Dolan 2014)

*I might trust that the information is well-protected, completely anonymous and requiring consent but one failing (and it doesn’t have to be my information specifically) and trust in the system is gone.*

Comment on: (Naughton 2014)

*… I’d love my data to be shared, with complete anonymity.*

Comment on: (Goldacre 2014)

The quotes above show that the underlying fear of parts of the public regarding anonymity is that anonymised data will be deanonymised and misused for re-identification. Comments show that such breach in confidentiality is anticipated and it is stated that this will result in decreasing levels of trust in the health system. That fear of data misuse is a major concern for individuals that aim to share their health data and is also confirmed in various surveys (Middleton et al. 2019; Milne et al. 2019; Melas et al. 2010). Further, a study on public attitudes towards commercial access of health data confirms the quotes above, as the study shows that a low understanding of the purpose of data aggregation and anonymisation exists among members of the public (IpsosMORI 2016). In addition, several studies identified that the public is often unaware of research processes and use of personal data in practice (Hill et al. 2013; Aitken et al. 2016; Wellcome Trust 2013; Ipsos 2016). On the opposite, those that already participated in research studies that involved sharing genetic health data, indicate that a relevant motivator for them is the possibility to receive individual results of the study (Goodman et al. 2018; Clayton et al. 2018; Brall et al. 2021). It is obvious, that individual results can only be provided when data is not fully anonymised, but individuals can be re-contacted. These findings from the US and Swiss context translate into our view that if sceptical parts of the public better understand how data is used and what the benefits of different levels of anonymity are, they might weigh the utility of individual results against the need of full anonymity.

In contrast to the quotes above calling for full anonymity, the scientific community stressed for some time in various research areas that maintaining complete anonymity will become increasingly difficult in the health system. Already in 2008, Lunshof et al. stated clearly that in the context of genetic research data donors “need to realize that they are potentially identifiable and that their privacy cannot be guaranteed” (Lunshof et al. 2008, 409). This split between a) the scientific debate and knowledge of the limits of anonymity and b) the wish of parts of the public for full anonymity opens a gap between science and the public which needs to be closed to protect public trust in data use.

### Initiating public engagement activities on the limits of anonymity

Since the scientific community discussed the limits of anonymity for a while (Kaye 2012; Dankar et al. 2019), it is worrying that parts of the public are still not fully informed about the limits of anonymity and what anonymity in the context of national data programmes and health research means. People with false expectations are at risk of feeling betrayed because of an unnecessary knowledge gap. This knowledge gap will become increasingly dangerous considering the development of current health research that depends particularly on the use of linked data, e.g. in genomics research or disease control. Due to the fact that society is increasingly revealing and using personal data in all areas of life, a reasonable understanding of what anonymity implies is advantageous. Further, if the public is well informed about anonymity and data linkage for research, the public is much more likely to support research using linked data sets and to build trust in research institutions (Aitken et al. 2016). Savage (2016) argues as anonymity is not the solution to privacy concerns due to the fact that full anonymity is impossible, it would be sensible to openly discuss and explain the benefits and risks concerning identification in the consent process and even before the public comes in close contact with research. Hence, there is a strong case for a large-scale information campaign to discuss with the public what anonymity means in the context of health research. When planning such an information campaign several questions need to be considered, foremost: What is the right format to address public concerns in a meaningful way? Who should lead such an endeavour? What are effective arguments?

**Fig. 1:**
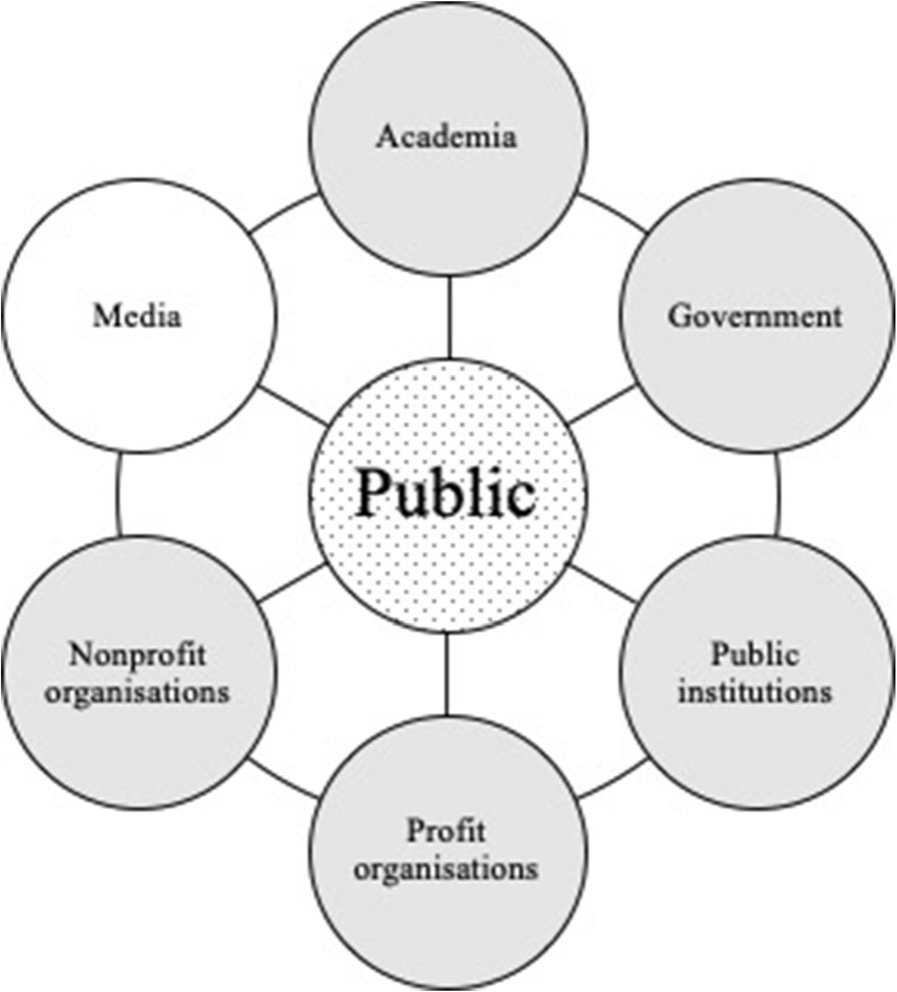
Network of actors that can engage with the public to discuss issues of anonymity. (Adapted from Brall et al. 2017; Brall 2018. Legend: Dotted = Data provider; Grey = Data user; White = Communication channel)

It is likely that the way forward to close this knowldge gap is a mixed approach including open public debate about concerns regarding anonymity and public engagement in health data governance processes, by which public participants are not only empowered, but also their health data literacy levels increase (Fischer 2012). Initiatives such as Understanding Patient Data can help to support public debate in a meaningful way (Understanding Patient Data 2020). Recent research on fair partnerships within the NHS England and the use of NHS patients’ data confirms that public participation in data governance processes to empower citizens is favourable towards increasing trust (Understanding Patient Data and Ada Lovelace Institute 2020). Ways to allow public participation are citizen juries or alternative health data governance models such as health data cooperatives where cooperative members have control about health data processes within the cooperative (Blasimme et al. 2018). Besides these possibilities to actively involve the public, health system representatives need to demonstrate how it is still possible to protect privacy despite not being able to provide full anonymity. Here, the responsibility to inform the public lies with the health data users. The information should directly address the concerns of the public in relation to anonymity and privacy. Moreover, the information should explain how privacy can be protected despite using the data in a not anonymized way. Answering the broader question ‘How is data kept safe?’ the Understanding Patient Data Initiative suggests removing identifying information, independent review processes, legal contracts and security standards (Understanding Patient Data 2021). To generate trust, ideally the conveyed information relates to a) comparative experiences to create a feeling of familiarity with the intended data use; b) present capabilities of the data user to show that s/he is capable to protect privacy despite not fully anonymizing data; and c) explain how privacy will be maintained in the future (Gille and Brall 2021; Gille et al. 2020). The nitty-gritty details of the information will remain country and context specific. In general, the efforts to inform the public about anonymity and thereby contribute to trust building should be distributed among all actors involved in health data uses (Gille et al. 2017). Every actor has to take responsibility to improve health data uses since “actions and circumstances of one stakeholder also affect a variety of other stakeholders within the network” (Brall 2018, 129). Adapted from our own research, such network of actors, as in Fig. 1, consists of several actors of the health data use network. The public, is at the center of the figure and provides health data. The grey shaded actors are health data users and the media (including social media) might not preliminarily be considered as a health data user, but is an important actor and communication channel that influences the public understanding of anonymity.

Actors that can shape the discussion around anonymity are academia, which performs research using health data and can contribute with expert opinion, and the government that shapes the regulatory landscape in which health data use takes place. Further actors are public institutions (including research and healthcare service institutions), for-profit organisations (such as pharmaceutical or health technology companies) and non-profit organisations (such as charities or professional associations). In order to support this endeavour on a broader scale, media should be involved as they are a major outreach instrument and it has been shown in the readership comments above that members of the public engage in this type of public fora. Also, previous research on health campaign communication strategies suggests to involve media outlets (Wakefield et al. 2010). For example, newspapers with a high national readership could potentially address these topics on a weekly basis by means of a health data series, where readers can ask questions and experts or health data users provide answers. Not only in the public health field in general, but also in health data use specifically, rethinking networks and their interdependencies to achieve sustainable structures is key. To inform people about anonymity during an informed consent process (where applicable) alone will not be sufficient as it is too late in terms of the overall recruitment process. Some people will have been put off from participating far earlier in the process before having participated in the informed consent process due to false understandings of anonymity. That said, for participants that engage in the consent process, the consent process remains an effective and important process to convey the limits of anonymity and to provide a clear description of the practical limitations of data anonymization.

In summary stakeholders have to jointly work together to involve the public:

- To openly discuss and explain the benefits and risks concerning identification.
- To empower citizens by engaging them in decisions about health data use.
- To demonstrate how privacy can be protected despite not being able to provide full anonymity.

## Conclusion

If the entire public is fully informed about the limits of anonymity, people can adapt their expectations towards anonymity and make better informed choices. This would not only benefit research, but also support public trust in the wider health system.

## Data Availability

All data generated or analysed during this study are included in this published article.
